# LATE RESULTS OF ESOPHAGOGASTRIC DEVASCULARIZATION AND SPLENECTOMY ASSOCIATED WITH ENDOSCOPIC TREATMENT IN PATIENTS WITH SCHISTOSOMIASIS

**DOI:** 10.1590/S0102-67202015000300013

**Published:** 2015

**Authors:** Walter De Biase da SILVA-NETO, Claudemiro QUIREZE-JÚNIOR, Thiago Miranda TREDICCI

**Affiliations:** Department of Surgery, Faculty of Medicine, Federal University of Goiás, Goiânia, GO, Brazil

**Keywords:** Schistosomiasis, Portal hypertension, Surgery, Esophageal and Gastric Varices

## Abstract

**Background:**

Schistosomiasis is endemic problem in Brazil affecting about three to four million people, and digestive hemorrhage caused by esophageal varices rupture is the main complication of the disease. Surgical treatment has become a therapeutic option, especially for secondary prophylaxis after at least one episode of bleeding. The surgical technique used by the vast majority of surgeons for the prevention of rebleeding is esophagogastric devascularization and splenectomy. Although with good postoperative results, rebleeding rate is significant, showing the need to follow-up endoscopy in all patients.

**Aim:**

To evaluate long-term results of patients submitted to esophagogastric devascularization and splenectomy and postoperative endoscopic treatment regarding esophageal varices caliber and rebleeding rates.

**Methods:**

A retrospective study of 12 patients underwent esophagogastric devascularization and splenectomy followed for more than five years.

**Results:**

All patients showed varices size reduction, and no patient had postoperative bleeding recurrence.

**Conclusion:**

Esophagogastric devascularization and splenectomy decreased significantly the esophageal variceal size when associated with endoscopic follow-up, being effective for bleeding recurrence prophylaxis.

## Abstract (pt)

**Racional:**

A esquistossomose acomete três a quatro milhões de pessoas no Brasil, sendo a hemorragia digestiva por ruptura das varizes esofágicas a principal complicação da doença. O tratamento cirúrgico é empregado como profilaxia secundária em pacientes com história de hemorragia prévia. A cirurgia mais utilizada é a desconexão ázigo-portal mais esplenectomia, técnica com bons resultados, porém com índice de recidiva hemorrágica considerável, fazendo necessário o seguimento endoscópico destes pacientes.

**Objetivo:**

Analisar a evolução tardia dos pacientes no que se refere à recidiva hemorrágica e ao comportamento das varizes esofágicas quando submetidos à desconexão ázigo-portal mais esplenectomia e tratamento endoscópico pós-operatório.

**Método:**

Foram avaliados retrospectivamente 12 pacientes submetidos à desconexão ázigo-portal mais esplenectomia com acompanhamento endoscópico pós-operatório maior de cinco anos.

**Resultados:**

Todos tiveram redução significativa do calibre das varizes e nenhum paciente apresentou sangramento pós-operatório.

**Conclusão:**

A desconexão ázigo-portal mais esplenectomia diminuiu significativamente o calibre das varizes esofágicas quando associada ao tratamento endoscópico pós-operatório. Este tratamento foi efetivo para a profilaxia da recidiva hemorrágica.

## INTRODUCTION

Schistosomiasis is an endemic problem in Brazil affecting about four million people 22 ; two to ten percent of infected individuals will develop the hepatosplenic form of the disease, characterized by periportal hepatic fibrosis, pre-sinusoidal portal hypertension and splenomegaly 13 .

Once installed the liver damage, there is no option for clinical treatment however, one of the characteristics of the disease is the preserved liver architecture, and especially, liver function 9 . Due to the preserved liver function, digestive hemorrhage caused by esophageal varices is the main and most fearsome complication of hepatosplenic shistosomiasis.

Studies have shown that gastrointestinal bleeding due to rupture of esophageal varices occur in 40% ¹ and it is the most threat to survival in patients with hepatosplenic schistosomiasis ²¹ with a mortality rate of 11.7% in the first episode of bleeding 12 . The importance of this disease regarding public health was demonstrated by a survey from the Ministry of Health between 1998 and 2009 that showed an annual mortality rate estimated to be between 0.2 and 0.34 deaths per 100.000 inhabitants ²³ .

With the improvement of drug and endoscopic therapy in bleeding esophageal varices control, surgical treatment has become a therapeutic option 19 , especially for secondary prophylaxis after at least one episode of bleeding. As the patient with schistosomiasis has preserved liver function, the surgical technique used in the vast majority of groups for the prevention of rebleeding is esophagogastric devasculatrization and splenectomy (EGDS). This procedure, leads to good results regarding bleeding control without the disadvantage of hepatic encephalopathy in the postoperative period.

Although leading to good postoperative results 8 , the rebleeding rate after EGDS is substantial, ranging from 6-29% 9 10 13 17 . However, Sakai et al, have shown a significant decrease in the postoperative bleeding rate when endoscopic therapy was annexed as an extension of postoperative follow-up 17 , showing the need of a follow-up endoscopy in patients submitted to EGDS. Nevertheless, there are few studies that evaluate the late results of this procedure.

The aim of this study was to evaluate long-term results with regard to rebleeding rate and behavior of esophageal varices of patients subjected to EGDS and postoperative endoscopic treatment.

## METHODS

This is a retrospective study of 12 patients submitted to EGDS between 1998 and 2005 and followed during long follow-up.

Patients with diagnosis based on epidemiological criteria, laboratory tests and confirmed by histopathological examination from liver biopsy performed as a routine during the surgery, with at least one previous episode of upper gastrointestinal bleeding due to rupture of esophageal varices, were evaluated. All surgeries were elective, at least 30 days since the last bleeding episode.

**FIGURE 1:**
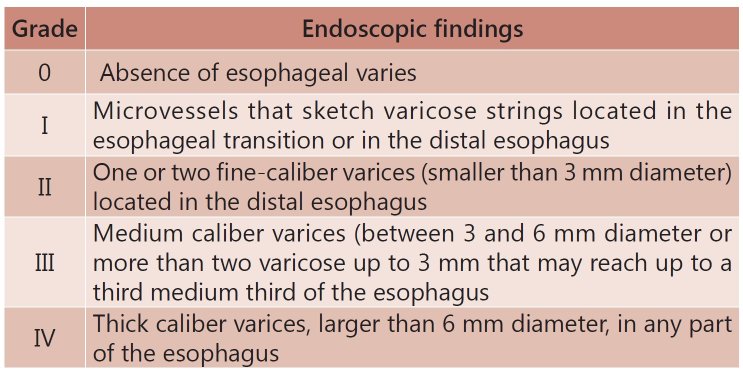
Esophageal varices evaluation according to Palmer e Brick criteria 15

Patients were classified according to the esophageal varices caliber based on Palmer and Brick classification 15 ( Figure 1 ).

Excluded criteria were: patients with other liver diseases, abnormal liver function tests, and positive serology for hepatitis B or C.

All patients were followed for more than five years by the general surgery team and also by the gastroenterology endoscopy group, being subjected to sclerosis of varicose veins with monoethanolamine maleate and 50% glucose, according to the routine of the service, anytime the patient had varicose veins. The postoperative interval between endoscopy reevaluation was one, three, six months and one year. After that, every other year if there was no need for another endoscopic intervention; however, the interval was individualized according to the therapeutic response.

## RESULTS

The mean age of patients was 36.6 (22-49) years and nine patients (75%) were male. All patients were monitored and the mean duration of postoperative follow-up was 11.9 (5-19) years. Late postoperative complications were not observed, neither related to surgery nor to endoscopic procedures. The average number of endoscopic exams per patient was eight (3-17). None of the patients lost follow-up.

In six patients (50%) the varices were eradicated. Endoscopic evaluation of the esophageal varices size is shown in Figure 2 . No patient had postoperative hemorrhage recurrence and there was no mortality during follow-up.

**FIGURE:**
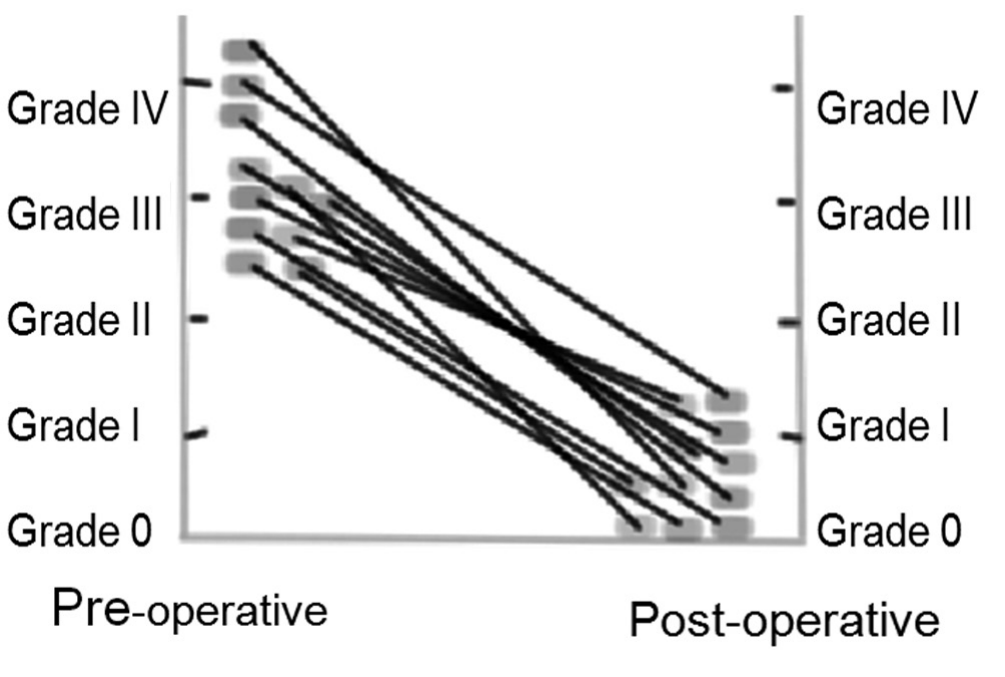
2 - Varices caliber evolution since pre-operative until last endoscopy

## DISCUSSION

The preferred surgery performed in most groups that deal with presinusoidal portal hypertension 2 3 4 10 , EGDS has the advantage of not triggering postoperative encephalopathy 16 , significant complication after porto-systemic shunts. The major drawback of EGDS is rebleedling that was significantly decreased since the endoscopic treatment (sclerotherapy) was introduced as a routine during follow-up 1 5 19 20 . However, there are few studies that evaluate the long-term results for these patients.

This study, with a mean follow-up higher than 10 years, showed that all patients had a significant reduction of their variceal size, in accordance of previous reported results with short 21 and long term follow-up 14 .

In all patients follow-up was superior to five years (in six cases greater than 10) with varices eradication in 50% of the cases, outcome similar to other studies as Ferraz et al with 52.7% and Makidissi et al with 85.7% of eradication, but with subsequent recurrence of varices in 56.6% of the cases in later examinations, achieving a final rate of 44% of eradication and lower than Batista-Neto et al, study that used a technical variant with a shorter follow-up (28 months) with 84,6% 1 9 13 .

The absence of rebleeding in this study is not consistent with others that found recurrences related to variceal bleeding ranging between 14.4 and 16.7% ¹ , ¹¹ 14 17 . This may have occurred because the meticulous endoscopic postoperative monitoring, which contributed to the eradication of esophageal varices or the maintenance of reduced caliber varices leading to a very low risk of rebleeding.

As shown by Cleva and colleagues^6^ and Evangelista-Neto et al. 8 there is varices venous pressure drop and a decrease in varices diameter after splenectomy and ligation of the left gastric vein 18 , favoring the intervention of the endoscopist. Due to the decrease in varicose veins diameter as a consequence of the decrease in its pressure, the risk of rebleeding is reduced.

In a late follow-up Cleva and colleagues demonstrated portal vein thrombosis in 55% of patients^5^, showing the need of other long term studies in this subject. 

Although a significant varicose veins decrease in diameter, the small sample of cases evaluated represents a limitation of this study. Therefore, further research with a larger number of patients is necessary for more conclusive results regarding a procedure that can prevent rebleeding, maintaining liver function, treat hypersplenism, and without inducing hepatic encephalopathy in individuals with schistosomotic portal hypertension with bleeding from esophageal varices rupture background.

## CONCLUSION

Esophagogastric devascularization and splenectomy decreased significantly the esophageal variceal size when associated with endoscopic follow-up, being effective for bleeding recurrence prophylaxis.
